# Use of cancer-specific yeast-secreted *in vivo *biotinylated recombinant antibodies for serum biomarker discovery

**DOI:** 10.1186/1479-5876-6-41

**Published:** 2008-07-24

**Authors:** Nathalie Scholler, Jennifer A Gross, Barbara Garvik, Lance Wells, Yan Liu, Christian M Loch, Arturo B Ramirez, Martin W McIntosh, Paul D Lampe, Nicole Urban

**Affiliations:** 1 Center for Research on Early Detection and Cure of Ovarian Cancer, School of Medicine, University of Pennsylvania, Philadelphia, Pennsylvania 19104, USA; 2 Molecular Diagnostics Program, Public Health Sciences, Fred Hutchinson Cancer Research Center, Seattle, Washington 98109, USA; 3 Complex Carbohydrate Research Center, University of Georgia, Athens, Georgia 30602, USA

## Abstract

**Background:**

Strategies to discover circulating protein markers of ovarian cancer are urgently needed. We developed a novel technology that permits us to isolate recombinant antibodies directed against the potential serum biomarkers, to facilitate the further development of affinity reagents necessary to construct diagnostic tests.

**Methods:**

This study presents a novel discovery approach based on serum immunoprecipitation with cancer-specific *in vivo *biotinylated recombinant antibodies (biobodies) derived from differentially selected yeast-display scFv, and analysis of the eluted serum proteins by electrophoresis and/or mass spectrometry.

**Results:**

Using this strategy we identified catabolic fragments of complement factors, EMILIN2, Von Willebrand factor and phosphatidylethanolamine-binding protein 1 (PEBP1 or RKIP) in patient sera. To our knowledge, this is the first report of a soluble form of PEBP1 in human. Independent evidence for ovarian cancer-specific expression of PEBP1 in patient sera was found by ELISA assays and antibody arrays with anti-PEBP1 antibodies. PEBP1 was detected in 29 out of 30 ascites samples and discriminated ovarian cancer sera from controls (p = 0.02). Finally, we confirmed by western blots the presence of a 21–23 kDa fragment corresponding to the expected size of PEBP1 but we also showed additional bands of 38 kDa and 50–52 kDa in various tissues and cell lines.

**Conclusion:**

We conclude that the novel strategy described here allows the identification of candidate biomarkers that can be variants of normally expressed proteins or that display cancer-specific post-translational modifications.

## Background

New biomarkers with the potential to detect disease early are critically needed for ovarian cancer. This study describes an innovative strategy to identify circulating proteins that signal disease. Our technology also permits isolation of recombinant antibodies directed against the potential biomarkers, which may facilitate the further development of affinity reagents necessary to build up diagnostic tests. Cancer-specific biotinylated recombinant antibodies (biobodies or Bbs [1]) were derived from a yeast-display recombinant antibody (single-chain Fragment variable or scFv) library [2] selected by multiple rounds of magnetic and fluorescence cell sorting for scFv that bind to sera from ovarian cancer patients (case-pool serum) but not to sera of healthy women (control-pool serum). Candidate biomarkers were immunoprecipitated from case-pool serum with cancer-specific biobodies and eluted for analysis. The quality of the procedure was evaluated by independent mass spectrometry experiments after tryptic digestion in-gel of the eluates separated by 1-D or 2-D gel electrophoresis, or in-solution directly from biobody eluates. One of the candidate markers identified was PEBP1, a member of the evolutionarily conserved phosphatidylethanolamine-(PE) binding proteins. As antibodies were commercially available, PEBP1 was further evaluated by ELISA test using an independent set of ovarian cancer ascites, by western blots on a panel of normal and tumor tissues and tumor cell lines, and by antibody arrays with a new set of case and control sera [3]. PEBP1 was found to be significantly elevated in patient sera on the antibody arrays as well as present in patient ascites by ELISA. PEBP1 is normally a basic cytosolic protein but our results suggest the existence in patient fluids of a soluble form of PEBP1 that is a serum marker for ovarian cancer.

## Methods

### 1) Overall strategy

**Figure 1:**
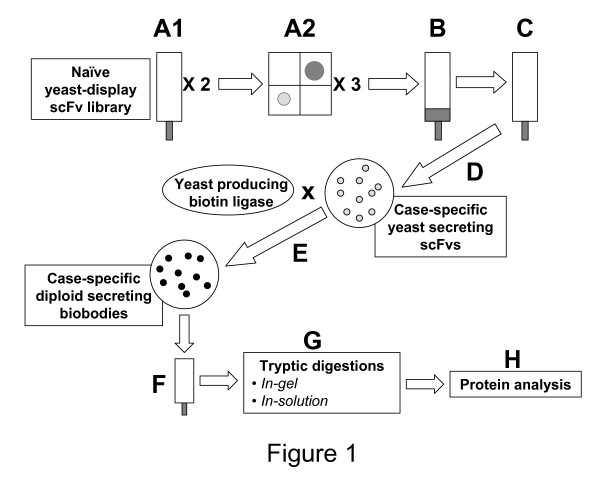
**Strategy overview**. A: Enrichments of a naïve yeast-display scFv library by (1) 2 magnetic sortings and (2) 3 flow sortings for the scFv that bound to 100 μg/ml of biotinylated case-pool serum. B: Depletions of the enriched sub-library by two magnetic depletions for the scFv that bound to 50 μg/ml of biotinylated control-pool serum. C: Enrichments for yeast that displayed scFv binding to 100 μg/ml of biotinylated case-pool serum, or that expressed c-myc tagged scFv. D: PCR amplification of case-pool serum-specific recognition sequences and cloning by gap repair in pTOR2 vector. E: High-throughput mating of yeast secreting scFv with yeast transformed with pTOR BIR to produce diploids that secrete biotinylated scFv, biobodies (Bbs). F: Immunoprecipitation of serum proteins with case-pool serum-specific biobodies and magnetic elution of immunoprecipitated case-pool specific serum proteins. G: Tryptic digestions in-solution or after in-gel separations of immunoprecipitated proteins. H: Mass spectrometry data acquisition and analysis.

The overall procedure to identify serum biomarkers is summarized in Figure 1. Cancer-specific biobodies were derived from yeast-display scFv selected by magnetic (fig. 1A1, B, C) and fluorescent (fig. 1A2) cell sortings for binding to sera from ovarian cancer patients but not to sera from healthy women. Recognition sequences of the selected yeast-display scFv were PCR amplified and cloned by gap repair into pTOR2 vector (fig. 1D) to produce case-specific yeast secreting scFv. The yeast secreting scFvs were mated with yeast carrying pTOR-BIR vector to generate case-specific diploid yeast that secreted *in vivo *biotinylated scFv (biobodies) (fig. 1E). Potential biomarkers were immunoprecipitated with cancer-specific biobodies, eluted (fig. 1F) and identified by mass spectrometry (fig. 1H) after tryptic digestion performed in-gel after protein electrophoresis or directly in-solution (fig. 1G).

### 2) Sera and ascites

For yeast-display scFv screening and immunoprecipitation, we used a discovery sample set of 22 sera, including 10 sera from serous ovarian carcinoma patients (stage IIIC n = 9 and stage IVB n = 1 with moderately n = 3 or poorly differentiated tumors n = 7) and 12 control sera (benign serous cysts n = 4, abnormal pap smear but normal ovaries n = 1, and healthy controls n = 7). Sera from ovarian cancer patients and sera from benign and healthy controls were pooled to create case- and control-pool sera, respectively. Sera were obtained from women who were fasting and under anesthesia. Diagnoses were made based on information in the clinical pathology report. All serum specimens were collected at Swedish Hospital, Seattle, WA, and were processed in similar fashion within 4 hours of collection. They were centrifuged at 1200 × g for 10 minutes, and the serum was aliquoted and frozen at -80°C. These pools were first depleted of abundant serum proteins using cibacron blue beads [4] and then biotinylated with the EZ-link sulfo-NHS LC biotin kit (Fisher Biotech, Fair Lawn, NJ) according to the manufacturer's instructions. Finally, biotinylated sera were dialyzed against 4 liters of phosphate buffered saline (PBS) (Fisher) overnight (ON) at 4°C and stored at -80°C. Protein concentrations were evaluated with the Nanoorange Protein Quantitation Kit (Invitrogen, Carlsbad, CA).

For validation purposes, a novel antibody array platform was probed with a validation sample set characterized in [3]. Briefly, the validation sample set included 65 independent serum samples from 31 serous ovarian cancer cases, including 2 that were diagnosed as stage IA, 2 diagnosed as stage IC, 1 stage IIA, 1 stage IIC, 2 stage IIIB, 18 stage IIIC, 4 stage IVA and 1 stage IVB, and from 34 controls, including healthy women (n = 16), women with benign ovarian disease (n = 10), and women undergoing surgery for gynecological conditions but who have histologically normal ovaries (n = 8). The age at collection ranged from 52 to 87 years old (mean 61.6; standard deviation 9.8) for the ovarian cancer cases and was very similar for the controls (range 42–86, with mean 60.9 and standard deviation 10.6). Samples from women undergoing surgery were obtained prior to surgery and chemotherapy.

For ELISA, 30 ascites from patients with ovarian cancers of different origins were used, including serous (21) diagnosed as stage IIIA (1), IIIB (2), IIIC (15), IVA (2) or unknown (1), mesodermal (mullerian) mixed tumor stage IVA (1), mucinous (2) recurrent or IIIC, adnocarcinomas (2) stage IVB or IIIC, clear cell stage IIIC (1), and endometrioid (3) stage IIIB, IIIC or IVA. Fluid was aspirated in the operating room at the time of surgery, and the cells were removed by centrifugation before the supernatant fluid was frozen at -80°C. Data on whether the cellular component was malignant or benign was recorded in the pathology report. Some western blots were loaded with cell lysates from cells removed by centrifugation from ascites fluids of six patients with serous ovarian cancer (metastatic disease n = 5; stage IIC n = 1; papillary n = 3; recurrent tubal primary n = 1).

### 3) Yeast media and antibodies

Yeast-display scFv were grown in synthetic selective medium containing 0.5% Casamino Acids (Fisher) and 1% penicillin/streptomycin (PS) (Gibco/Invitrogen Corporation, Carlsbad, CA), and induced in selective medium supplemented with 2% galactose, 2% rafinose, 0.1% dextrose, and 1% PS as previously described [1,5]. Anti-c-myc mouse monoclonal antibody (mAb) 9E10 was purchased from Santa Cruz Biotech (Santa Cruz, CA); Alexa Fluor^® ^488 F(ab')2 fragment of goat anti-mouse IgG (H+L) (488 anti-mIg) was purchased from Invitrogen and phytoerythrin-labeled streptavidin (SA-PE) was purchased from Becton Dickinson (Pharmingen, San Diego, CA).

Three antibodies against PEBP1 were purchased from Abgent (San Diego, CA) and Millipore (Fisher). The purified rabbit polyclonal antibodies (pAb) from Abgent were raised against synthetic peptides selected from the conserved central PE-binding region of human PEBP1 (center pAb) or from the N-terminal region (N-term pAb). Anti-PEBP1 pAb from Millipore was raised against a GST-fused full-length rat PEBP1 recombinant protein and used for ELISA and western blots. Horseradish peroxidase (HRP)-conjugated anti-rabbit Ig antibodies used for ELISA assays were purchased from Promega (Madison, WI).

### 4) Differential screening of a yeast-display library for scFv that bind preferentially to ovarian cancer sera and generation of case-serum-specific biobodies

The yeast-display scFv library created by the Pacific Northwest National Laboratory (PNNL) was used [2]. This library was constructed from human naïve B-lymphocytes and its size is 2 × 10^9 ^yeast-display scFv. Incubations of yeast-display scFv with biotinylated human sera were carried out with 10^8 ^to 10^9 ^yeast per ml in PBE [PBS supplemented with 0.5% bovine serum albumin (Sigma-Aldrich, St. Louis MO) and 4 mM EDTA (Promega)] for 30 minutes at 4°C. Sublibraries of yeast-display scFv that bound to biotinylated case-pool serum were derived from the entire yeast-display scFv library [2] first by magnetic sorting using streptavidin or anti-biotin magnetic beads (Miltenyi, Auburn, CA) and MACS LS separation columns (Miltenyi), and then by fluorescent cell sorting using the BD FACSAria™ cell sorter (BD Biosciences Immunocytometry Systems, San Jose, CA) as previously described [1,5]. Next, the enriched sublibraries were depleted for the scFv that bound to the control-pool serum by magnetic sorting, using anti-biotin magnetic beads (Miltenyi) and MACS LD separation columns (Miltenyi) according to the manufacturer's instructions. The effluents containing yeast-display scFv that did not bind to control-pool serum were subsequently enriched one more time for scFv binding to case serum. In some experiments the effluents were enriched for c-myc-expresser yeast with anti-c-myc magnetic beads (Milenyi) to remove yeast that failed to bind to the control column because they lacked scFv expression. The recognition sequences of the yeast-display scFv that demonstrated a preferential binding to case- over control-pool serum were amplified by PCR and cloned in yeast by gap repair using co-transformation with pTOR2 vector [1] for production of tagged, yeast-secreted scFv. We previously described a method to *in vivo *biotinylate scFv on a specific target site that is separated from scFv binding site by a flexible linker (IgA hinge) [1]. Using this method, the scFv binding site is not compromised by random biotin-binding to any available lysines. Furthermore, scFv form tetramers of high affinity in presence of streptavidin, which improves their conformational stability and makes it possible to use them as detector reagents in diagnostic tests [1,5,6]. To *in vivo *biotinylate the scFv, scFv-secretor yeast were mated with yeast carrying a biotin ligase fused to golgi-localization signals encoded by pTOR BIR [1] to generate diploid yeast that secrete case serum-specific biobodies. Finally, biobodies were purified on HIS-Select™ Nickel Affinity Gel (Sigma-Aldrich) and used to immunoprecipitate candidate biomarkers from serum pools (Fig. 1F).

### 5) Immunoprecipitation of case specific-serum proteins with biobodies (Bbs)

Eight hundred μl of serum from case and control pools were incubated with 8 μg of biobodies ON at 4°C with rotation. Then 50 μl of streptavidin-coated micromagnetic beads (μMACS Streptavidin Kit, Miltenyi) were added to the serum pool/biobody complexes, incubated 1 hour at 4°C with rotation and loaded onto μMACS columns (Miltenyi). Immunoprecipitated serum proteins were eluted in loading buffer according to manufacturer's instructions for protein electrophoresis or with 8 M Urea (Amersham Biosciences, Piscataway, NJ) for shotgun mass spectrometry.

### 6) Protein electrophoresis

Five μl of eluate in loading buffer were separated on one dimensional (1-D) (NuPAGE^® ^Gel, 4–12%, 10 wells, Invitrogen) or on two dimensional (2-D) protein gels. For 2-D gels, immunoprecipitates from case- and control-pool sera were treated with the 2-D Cleanup Kit (Amersham) according to manufacturer's instructions, protocol A. The precipitated samples were resuspended in 250 ml rehydration buffer, 8 M urea (Amersham), 2% w/v CHAPS (Calbiochem, Darrnstadt Germany), 0.5% IPG Buffer pH3 to pH10 NL (GE Healthcare, Piscataway, NJ), 0.002% bromophenol blue (GE Healthcare), 0.1% dithiolthreotol (DTT) (Fisher), centrifuged briefly to remove particulate matter, and transferred into isoelectric focusing chambers. A 13 centimeter Immobiline Dry Strip pH3 to pH10 NL was immersed in each sample, overlain with IPG Cover Fluid (GE Healthcare) and focused in an Ettan IPGphor™ Isoleclectric Focuser according to the following protocol: Rehydration 12 hours at 50 volts per strip, step to 500 volts 1 hour, gradient to 1000 volts 1 hour, gradient to 8000 volts 2.5 hours, hold 8000 volts 8 hours, step to 500 volts for 2 or more hours.

2-D electrophoresis was performed on an Ettan IPGphor™ Isolelectric Focusing System (Amersham) accordingly to the manufacturer's instructions. Two vertical 14 × 13 centimeters × 1.0 millimeter 10% acrylamide gels were prepared using 30% acrylamide bis 29:1 (Bio-Rad Laboratories, Hercules, CA). The gels also contained 0.19 M Tris HCl pH 8.8, 0.075% SDS, 0.025% TEMED (GE Healthcare) and 0.06% ammonium persulfate (GE Healthcare). The gels were overlaid with running buffer: 250 mM Tris base, 1.92 M glycine, 1% SDS. The focused Immobiline Dry strips were washed gently in nanopure water and incubated separately at room temperature for 15 minutes with gentle agitation in 10 ml of 50 mM Tris HCL pH8.8, 6 M urea, 30% glycerol (Fisher), 2% SDS, 0.002% bromophenol blue containing 100 mg of DTT. The strips were then gently washed again with nanopure water and incubated for 15 minutes at room temperature (RT) in 10 ml of the same solution but with 250 ml of iodoacetimide (GE Healthcare) instead of DTT. Each strip was laid edgewise on the surface of an acrylamide gel and the running buffer was poured off and replaced with running buffer containing 0.5% agarose (Fisher Scientific), 0.5% bromophenol blue to completely fill the space above the gels. The gels were placed in Hoefer SE 600 Ruby Electrophoresis Boxes (Amersham) and electrophoresed with Tris Glycine SDS running buffer at 15 milliamps per gel for 30 minutes and then at 25 milliamps per gel until the dye front reached the bottom edge.

Protein were visualized with SilverQuest™ Silver Staining Kit (Invitrogen) and bands/spots that were differentially expressed by case vs. control samples were excised with a clean razor blade from both case and control sample electrophoresis (1D gel) or from case sample only (2D gel) and identified by mass spectrometry and the Computational Proteomics Analysis System (CPAS) [7].

### 7) Mass spectrometry data acquisition and analysis

For in-solution digestion of proteins (e.g., as described in detail by [8]), samples were prepared using standard approaches. Briefly, samples from control and case eluted in 8 M urea were diluted to 1 M urea in 40 mM ammonium bicarbonate, reduced with DTT, alkylated with iodoacetamide, and digested ON with sequencing grade trypsin (Promega). The resulting peptides were acidified and desalted using C18 spin columns (The Nest Group, Southborough, MA). Peptides were resuspended in 0.1% formic acid and nitrogen bomb loaded onto an in-house packed C18 (75 μm by 9 cm) reverse phase column/emitter (New Objectives, Woburn, MA).

In-solution digested peptides were evaluated using an LTQ mass spectrometer (ThermoFinnegan). Peptides were eluted with a 150 minute linear gradient of increasing acetonitrile in 0.1% formic acid. The tandem mass spectrometer was configured so that one full MS spectrum was collected followed by collision-induced fragmentation and MS/MS spectra acquisition on the 8 most intense ions. Dynamic exclusion was set at 2 for 30 seconds. Peptides were identified using bioworks/TurboSequest (version 3.2) using an NCBI human database allowing only for strict trypsin digestion. For filtering of the data, an inverted (decoy) database was used to calculate the false-discovery rate. Data reported are filtered at a false-discovery rate below 1% at the peptide level.

1-D and 2-D gel analysis were evaluated following tryptic digest using high-resolution mass spectrometry (LTQ-FT, or LTQ, Thermo Finnegan). All data were evaluated using a freely available version of CPAS 2.0 [7], where raw data was converted to mzXML form and searched using the X!Tandem [9] search engine configured with k-score [10] against IPI version 20060111, then evaluated using peptide prophet [11]. All proteins containing at least two peptides exceeding 0.95 detection probability, exceeded minimal ionization percentages (15%) and were not common contaminants (e.g., Keratin) were considered for further analysis.

### 8) ELISA

ELISA immunoassays were performed in Nunc Amino™ plates (Nunc, Rochester, NY) coated with ascites from ovarian cancer patients diluted 1:1,000 in bicarbonate buffer (Carbonate-Bicarbonate Buffer Capsules, Sigma-Aldrich) for 1 hour at RT. All other incubations were done in PBS supplemented with 0.05% Tween (Fisher) (PBST), at RT for 30 min with gentle agitation. After three PBST washes, ascites-coated wells were incubated with anti-PEBP1 antibodies; pAbs were diluted to 250 ng/ml. After three washes with PBST, wells were incubated with HRP-anti-rabbit Ig diluted 1:2000. Finally colorimetric signals were generated with TMB One Solution (Promega), stopped with 1N H_2_SO_4 _(Acros Organics USA, Morris Planes, NJ) and read at 450 nm on a SpectraMax M5/M5^e ^Microplate Reader (Molecular Devices, Sunnyvale, CA).

### 9) Western blots

Western blots were performed as previously described [1] with the following modifications: lanes were loaded with 5 μg of PEBP1-GST fusion protein (46–49 kDa) (Abnova Corporation, Taipei City, Taiwan), 5 μg of ascites cell lysates, 15 μg of tissue lysates or 5 μg of cell lysates.

### 10) Antibody arrays

A novel high-density microarray platform that has the capacity to hold more than 18,000 binding agents (here we used antibodies directed against candidate biomarkers) was created, validated as previously described [3] and probed here with ovarian cancer and control sera. Briefly, analysis of hybridization was performed for fold-change of signal (case or control compared to reference channel). Fold-change of signal was calculated as log Rc/Gc; where Rc is red corrected (Cy5 spot signal minus background) and Gc is green corrected (Cy3 spot signal minus Cy3 background). Following normalization, triplicate spots were summarized using their median and classification was performed using logistic regression predicting case status based on log (fold-change), and including covariates adjusting for day and batch effects used in the fabrication and hybridization of the arrays. The probability score (p-value determined by Wilcoxon sign-rank testing) corresponding to the coefficient of the protein (e.g., log ratio) was used as the per-antibody significance measure.

## Results

### 1) Yeast-display library enrichment and depletion

**Figure 2:**
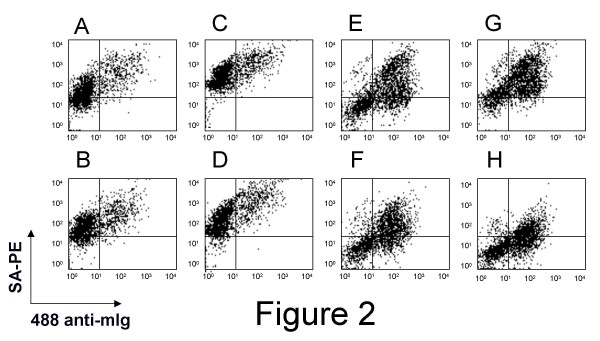
**FACS of yeast-display scFv library enriched for case-specific scFv**. A naïve yeast-display scFv library was enriched by magnetic and fluorescent cell sortings for scFv that bound to case-pool serum. The enriched yeast-display scFv were labeled with anti-c-myc mAb and 25 μg/ml (A,B,E,F) or 100 μg/ml (C,D,G,H) of biotinylated case (A,C,E,G) or control (B,D,F,H) serum pools, before (A-D) or after depletion by magnetic sorting (E-H). Binding signals were detected as shown with the secondary antibody 488-alexa anti-mouse Ig (488 anti-mIg) and PE-labeled streptavidin (SA-PE).

A yeast-display scFv library [2] was first enriched for protein serum-specific scFv with biotinylated case-pool serum by magnetic and flow sortings (fig. 1A1–2), until more than 95% of the selected yeast expressed scFv that bound to biotinylated case-pool serum (fig 2A,C, upper right quadrants). But the selected yeast also bound to the biotinylated control-pool serum (fig 2B,D, upper right quadrants). Thus, various regimens of depletion for yeast that bound to control-pool serum were tried in independent experiments. Immobilization of albumin-depleted control-pool serum on a plastic plate for panning gave poor results, and depletion experiments using biotinylated albumin-depleted control-pool serum for fluorescent cytometry sorting biased the selection towards yeast that did not express scFv (non-expresser yeast) (data not shown). The best results were obtained with magnetic sorting using depletion columns (see Experimental Procedures) for retrieval of control serum-binding scFv. Although depletion with control-pool serum at concentrations of 100 μg/ml biased the selection toward non-expresser yeast (data not shown), depletion with 30 μg/ml of control-pool serum yielded a population of yeast-display scFv that strongly bound to 100 μg/ml of case-pool serum (fig. 2G, upper right quadrant) and weakly bound to 100 μg/ml of control-pool serum (fig. 2H, upper and lower right quadrants). Of note, a discrete subset of this selected population of yeast-display scFv bound to mesothelin recombinant antigen (meso-Ig) [12], a known biomarker for ovarian cancer [13-15], while no binding to meso-Ig was distinguishable using the naïve yeast-display scFv library (data not shown). Altogether, these data suggested that the yeast-display scFv subtractive library selected with 100 μg/ml of ovarian case-pool serum and depleted with 30 μg/ml of control-pool serum was significantly enriched for scFv binding preferentially to serum proteins from ovarian cancer patients. This yeast-display scFv was used for the generation of cancer-specific biobodies (Bbs) as described in Experimental Procedures.

### 2) Identification of biomarkers by immunoprecipitation, electrophoretic separation by 1D or 2D gels, and tandem mass spectrometry after in-gel tryptic digestion

**Figure 3:**
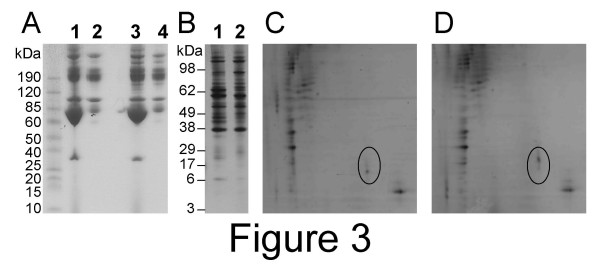
**Electrophoretic protein separations**. A: Control- (lanes 1,2) and ovarian cancer-pool (lanes 3,4) serum samples were separated by protein electrophoresis before (lanes 1,3) or after (lanes 2,4) depletion for abundant proteins by cibacron blue and detected by coomassie blue straining. B-C: Control- and ovarian cancer-pool serum depleted for abundant sequences were immunoprecipitated with biobodies selected for specific binding to case-pool serum. The products of elution from control sera (B lane 2, and C) and ovarian cancer (B lane 1, and D) were separated by 1-D (B) or 2-D (C,D) protein electrophoresis and detected by silver staining. In the 2-D gels, the immunoprecipitates were focused over a pH3 to pH10 range and run on a 10% acrylamide gel. The circle indicates the region where PEBP1 was found in patient serum (D) but not in control serum (C).

Albumin-depleted case- and control-pool sera were incubated with Bbs and streptavidin magnetic beads. The Bbs/serum-protein/magnetic bead complexes were retrieved using the μMACS system. Proteins were eluted and separated by electrophoresis and then visualized by silver staining (fig. 3B). Despite a large number of common bands between control and cancer, six bands were consistently found to be differentially immunoprecipitated on the 1-D gel. A 96 kDa-band was preferentially immunoprecipitated from control-pool serum (fig. 3B, lane 2) while five bands (22, 36, 42, 45 and 65 kDa) were preferentially immunoprecipitated from case-pool serum (fig. 3B, lane 1). On the 2-D gels, several spots migrated differentially between case and control, including the spot shown in Figure 3C,D. The five bands preferentially immunoprecipitated from case-pool serum (fig. 3B, lane 1), the corresponding fragments from the control-pool serum immunoprecipitation, and the spot shown in Figure 3D were excised from the gels and identified by mass spectrometry after in-gel tryptic digestion. In the 1D gels, the bands preferentially immunoprecipitated from case-pool serum yielded the complement C3 precursor (22 kDa-, 42 kDa- and 65 kDa-bands), the complement 4A precursor (36 kDa- and 65 kDa-bands), SERPINA1 (42 kDa-band) and the complement 4B precursor (45 kDa-band). Interestingly, the peptide coverage was noticeably different from one band to another. In the 22 kDa-band, peptides mapped over 6% of the complement C3 precursor and only in the C-terminal fraction, but in the 45 kDa- and 65 kDa-bands they mapped over 19% and 32% of the whole protein, respectively (Tables 2 and 3). Similarly, the peptide coverage on the complement 4A precursor in the 36 kDa-band was of 5% of the C-terminal portion only, while in the 65 kDa-band it was of 10% over the whole protein. Finally, in the 2-D gels (spot indicated in Figure 3D, apparent molecular weight of 22 kDa) we identified two unique peptides KLYTLVLTDP DAPSR and KGNDISSGTVLSDYVGSGPPK that were found by peptide prophet to map in the central PE-binding region of the protein PEBP1 with a probability 0.999 and 0.983, respectively. These two peptide hits met our criteria for robustness because they resulted from CPAS analysis, exceeded 0.95 detection probability, had at least 15% of minimal ionization percentages and were not common contaminants.

### 3) Identification of biomarkers by immunoprecipitation and tandem mass spectrometry after in-solution tryptic digestion (shotgun MS)

Proteomics analysis (LC-MS/MS) was also performed directly without electrophoresis on eluted proteins from the immunoprecipitations of control- and case-pool sera with case-specific biobodies. In the case sample, 60 peptides representing a total of 24 proteins were identified (Table 1), while no proteins or peptides were identified in the control group. Many of the assigned proteins were antibody fragments and complement associated proteins. These proteins were highly enriched indicated by the fact that only two peptides for human serum albumin and for haptoglobin, two of the most abundant sera proteins, were detected. In addition, this screen also identified EMILIN-2 and Von Willebrand factor.

### 4) Validation of PEBP1 by ELISA assay, western blots and antibody array

**Figure 4:**
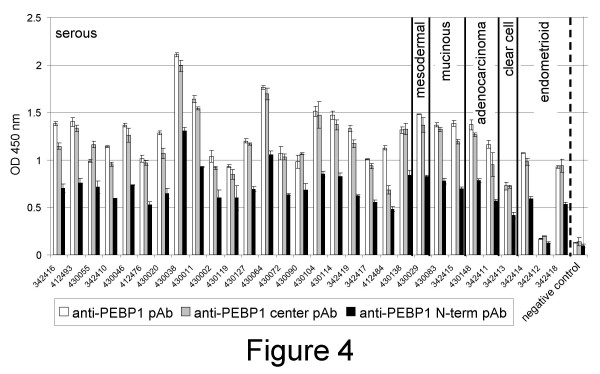
**PEBP1 detection in ascites fluids**. Ascites of patients with ovarian cancers from serous mesodermal, mucinous, adenocarcinoma, clear cell or endometrioid origin (as shown) were diluted in binding buffer, coated on plastic wells, and detected with polyclonal antibodies made to the center (pAb, white bars) or N-terminal (pAb, gray bars) region of PEBP1 or with a pAb made to the whole recombinant protein (black bars). Samples were tested in triplicates. Averages are shown. Wells coated with binding buffer were used as negative controls.

By detection ELISA assays, polyclonal antibodies (pAb) against the N-terminal (anti-PEBP1 N-term pAb), the center of PEBP1 (anti-PEBP1 center pAb), and the whole protein (anti-PEBP1 pAb), detected the presence of PEBP1 in 29 out of 30 ovarian cancer ascites, including ascites from ovarian cancers of non-serous origin (fig. 4). The only ascites that did not contain PEBP1 was from a patient with endometrioid ovarian cancer (fig. 4).

**Figure 5:**
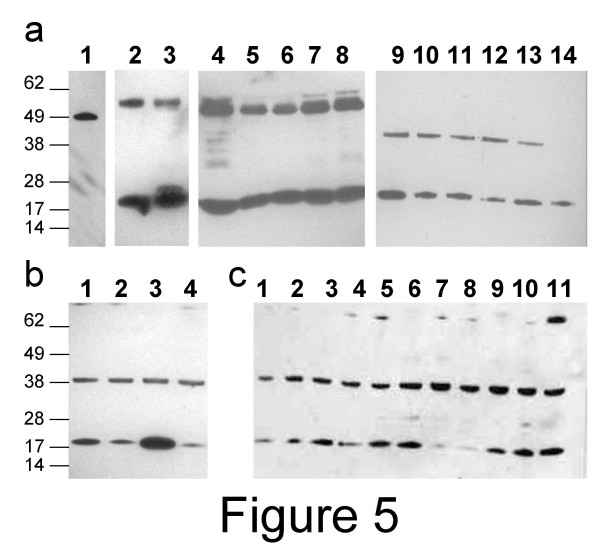
**PEBP1 detection in ascites cells, normal and tumor tissues, and tumor cell lines**. Cell lysates from normal tissues [ovary (a2), testis (a3)], tumor tissues [ovary (a4), prostate (a5), testis (a6), cervix (a7), uterus (a8)], ovarian cancer ascites cells (a9–14), prostate tumor cell lines (b1: BPM1; b2: RWPE-1; b3 VCap; b4: DU145) and ovarian tumor cell lines (c1: A1847; c2: A2780; a3: CaOv3; a4: ES2; a5: Hey; a6: IGROV1; a7: OvCar3; a8: OvCa5; a9: OvCar10; a10: Ov-90; a11: PEO-1) were diluted in PBS and separated by electrophoresis. After blotting, membranes were hybridized with anti-PEBP1 pAb. The signal was detected with HRP-labeled anti-rabbit Ig. As control of antibody specificity, lane a1 was loaded with a recombinant PEBP1 antigen fused to GST (49 kDa) (Abnova Corporation, Taipei City, Taiwan).

By western blot, anti-PEBP1 pAb detected a small band of 21–23 kDa in all tested samples (fig. 5a) but also a band of 50 kDa in tissue lysates (fig. 5a2–8) and a band of 38–40 kDa in five out of six ascites cells (fig. 5a9–13), and all prostate and ovarian cell lines tested (fig. 5b and data not shown).

The overall comparison between case and control sera was performed on a protein array platform using anti-PEBP1 center pAb and an independent set of ovarian cancer and control sera. This analysis generated preferential binding of anti-PEBP1 antibody to case serum with a p-value of 0.02, demonstrating that the presence of PEBP1 in serum is able to differentiate ovarian carcinoma from control sera. Anti-PEBP1 center pAb ranked 17 out of 338 full length antibodies and performed better than 4 out of 8 anti-CA125 mAbs [3] in preferential binding to the serum of individual cancer patients, suggesting that PEBP1 is a marker for ovarian cancer.

## Discussion

We have developed an innovative strategy to identify novel serum biomarkers for ovarian carcinoma using case-specific yeast-secreted *in vivo *biotinylated recombinant antibodies (biobodies or Bbs) to immunoprecipitate potential biomarkers. Candidate biomarkers were identified using a discovery sample set of 22 sera and validated on an independent sample set of 65 sera and 30 ascites fluids. Immunoprecipitated proteins from case- and control-pool sera (eluates) were separated by 1-D and 2-D gel electrophoresis and differentially expressed bands or spots were analyzed by mass spectrometry analysis, or eluates were directly analyzed by shotgun mass spectrometry after in-solution tryptic digestion. Shotgun mass spectrometry confirmed the results of 1-D gel by identifying multiple fragments of the complement cascade and in addition found EMILIN2 and Von Willebrand factor. Interestingly, EMILIN2 has been found to play a role in trophoblast invasion of the uterine wall and is enriched at the level of mRNA expression in ovarian serous tumors [16,17], and Von Willebrand factor has been associated with multiple cancers [18]. Analysis by 2-D gel identified a member of the PEBP family, PEBP1.

We did not identify well-known ovarian cancer markers such as CA125 [19], HE4 [20,21] or mesothelin [13]. However, a discrete subset of the case-selected yeast-display scFv bound to mesothelin, which suggests that despite the presence of anti-mesothelin scFv in the case-specific biobody pool, the efficient immunoprecipitation of mesothelin serum protein was not achieved. Antigen immunoprecipitation depends on the epitopes recognized by the antibody rather than on its overall antigen specificity [22]. Thus, this result is not surprising and implies that biomarker discovery using this approach is biased by the epitope recognition of the selected scFv. Generation of large scFv libraries from ovarian cancer patient lymphocytes could improve the discovery rate using this method.

We identified several complement factors by both 1D-gel and shotgun mass spectrometry, but they were shorter than expected for full length proteins. The complement (C) system consists of a set of plasma and membrane-bound proteins including C3 that protects the organism against invading microbes and abnormal cells. The regulation of C3 cleavage is critical for C function and is mediated by several molecules, including the membrane-bound proteins CD35, CD46 and CD55 [23-25] and the plasma proteins factor H (FH) and FH-like protein 1 (FHL-1) [26,27]. FH expression or binding contributes to protection against C of some pathogenic microbes (e.g., *Streptococcus pyogenes, S. pneumoniae, Neisseria gonorrheae, Borrelia sp*) [28-30] and, importantly, of tumor cells [31]. In addition, FH and FHL-1 are abundantly present in ovarian carcinoma ascites and primary tumors [32], and ovarian tumor cells have been reported to bind both FH and FHL-1 and to promote factor I-mediated cleavage of C3b to inactive iC3b [33]. Thus, the identification of small fragments of C factors in ovarian cancer patient sera may be due to a cancer-specific catabolic activity that inactivates C factors and protects tumor cells from the innate immune response.

By 2-D gel analysis, we identified several spots that migrated differentially between case and control serum pools. One of them was identified as PEBP1, also known as Raf-1 kinase inhibitor protein (RKIP) and hippocampal neurostimulating peptide precursor (HCNP). PEBP1 is a member of the phosphotydalethanolamine (PE)-binding proteins (PEBPs) that are 21 to 23-kDa basic cytosolic proteins [34] with preferential *in vitro *affinity for PE, a component of the inner leaflet of the plasma membrane [35,36]. RKIP/PEBP1 has been shown to disrupt the Raf-1-MEK1/2 [mitogen-activated protein kinase-ERK (extracellular signal-regulated kinase) kinase-1/2]-ERK1/2 and NF-κB signalling pathways with physical interaction with Raf-1-MEK1/2 and NF-κB-inducing kinase of TGFb-activating kinase-1 respectively, thereby abrogating the survival and anti-apoptotic properties of these signalling pathways. By regulating cell signalling, growth and survival through its expression and activity, RKIP is considered to play a pivotal role in cancer, regulating apoptosis induced by drugs or immune mediated stimuli [37]. Overexpression of PEBP1-RKIP sensitizes tumor cells to chemotherapeutic drug-induced apoptosis [37]. PEBPs belong to an evolutionarily conserved family of proteins represented in all three major phylogenetic divisions [38-42] with pivotal biological functions [11,42,43]. There are 13 identified mammalian PEBP sequences with highly conserved central region believed to be essential for PEBP function and binding to G proteins. No obvious secretion signal is present in the amino acid sequences, but in nematodes PEBPs are found to be part of the secreted cell surface proteins and protect against host immunological responses [44].

A polyclonal antibody directed against the conserved PE-binding sequence of PEBP1 could accurately (p = 0.02) discriminate between ovarian cancer and control sera on antibody arrays. This antibody as well as two other polyclonal antibodies directed against PEBP1 N-terminal region or the whole PEBP1 protein could also detect large amounts of protein in 29 out of 30 ovarian cancer patient ascites. Finally, the polyclonal antibody against the whole protein could detect larger fragments (38 and 50–52 kDa) in tissues, ascites cells and tumor cell lines, in addition to the 21–23 kDa fragment. Although we cannot exclude that the presence of PEBP1 in ovarian patient fluids could result from tumor cell death and degradation, altogether our results suggest that additional variants of PEBP1 exist in ovarian cancer patient fluids. It is conceivable that PEBP1 variants could play a role in tumor growth by interfering with PEBP1 inhibitory functions in survival and anti-apoptotic signalling pathways.

## Conclusion

Our results strongly suggest that PEBP1 variants are present in the sera and ascites of ovarian cancer patients. To our knowledge, PEBP proteins have never been described in ovarian cancer sera, although PEBP1 was recently identified in cell culture supernatants despite its lack of secretion signal [43,45]. In conclusion, using a novel type of biotinylated recombinant antibodies that we call biobodies, we identified complement catabolitic products, EMILIN2, Von Willebrand factor and PEBP1 or a related protein, as candidate markers for ovarian carcinoma.

## List of abbreviations used

1-D gel: 1-dimensional gel; 2-D: 2-dimensional gel; Bbs: biobodies; CPAS: Computational Proteomics Analysis System; DTT: dithiolthreotol; EDTA: ethylenediaminetetraacetic acid; ELISA: Enzyme-Linked ImmunoSorbent Assay; FACS: Fluorescence analysis cell sorting; H_2_SO_4_: Sulphuric Acid; HRP: Horseradish peroxidase; Ig: immunoglobulin; M: molar; mAb: mouse monoclonal antibody; mIg: mouse immunoglobulin G; ml: milliliters; pAb: polyclonal antibodies; PBE: PBS supplemented with 0.5% bovine serum albumin; PBS: phosphate buffered saline; PBST: PBS supplemented with 0.05% Tween; PEBP1: phosphatidylethanolamine-binding protein 1; PE: phosphatidylethanolamine; PCR: polymerase chain reaction; PS: penicillin/streptomycin; RT: room temperature; SA-PE: phytoerythrin-labeled streptavidin; scFv: single chain Fragment variable; SDS: sodium dodecyl sulfate.

## Competing interests

The authors declare that they have no competing interests.

## Authors' contributions

NS designed the study, initiated library screenings and antigen immunoprecipitation with biobodies, participated in the analysis of the data and wrote the manuscript. JAG completed library screenings and immunoprecipitations, and performed ELISA validations. BG did the electrophoresis separations by 1D- and 2D-gels. LW identified novel biomarkers by shotgun MS. CML and ABR performed the antibody arrays. YL, MWM, LW and PDL analyzed and controlled the data. NU participated in all steps of the study, including conception, design, coordination, data analysis and writing.
